# Process and formulation variables in the preparation of injectable and biodegradable magnetic microspheres

**DOI:** 10.1186/1477-044X-5-2

**Published:** 2007-04-04

**Authors:** Hong Zhao, Jeffrey Gagnon, Urs O Häfeli

**Affiliations:** 1 Faculty of Pharmaceutical Sciences, The University of British Columbia, 2146 East Mall, Vancouver, B.C. V6T 1Z3, Canada

## Abstract

The aim of this study was to prepare biodegradable sustained release magnetite microspheres sized between 1 to 2 μm. The microspheres with or without magnetic materials were prepared by a W/O/W double emulsion solvent evaporation technique using poly(lactide-co-glycolide) (PLGA) as the biodegradable matrix forming polymer. Effects of manufacturing and formulation variables on particle size were investigated with non-magnetic microspheres. Microsphere size could be controlled by modification of homogenization speed, PLGA concentration in the oil phase, oil phase volume, solvent composition, and polyvinyl alcohol (PVA) concentration in the outer water phase. Most influential were the agitation velocity and all parameters that influence the kinematic viscosity of oil and outer water phase, specifically the type and concentration of the oil phase. The magnetic component yielding homogeneous magnetic microspheres consisted of magnetite nanoparticles of 8 nm diameter stabilized with a polyethylene glycole/polyacrylic acid (PEG/PAA) coating and a saturation magnetization of 47.8 emu/g. Non-magnetic and magnetic microspheres had very similar size, morphology, and size distribution, as shown by scanning electron microscopy. The optimized conditions yielded microspheres with 13.7 weight% of magnetite and an average diameter of 1.37 μm. Such biodegradable magnetic microspheres seem appropriate for vascular administration followed by magnetic drug targeting.

## Background

Controlled release parenteral nanospheres and microspheres made from biodegradable polymers such as poly (lactide-co-glycolide) (PLGA) have been widely investigated for a variety of therapeutic agents. PLGA polymers have an excellent record of biocompatibility, biodegradability and non-immunogenicity [1-3]. PLGA polymers have also been used in the preparation of controlled release delivery systems, such as Lupron Depot^® ^(TAP Pharmaceutical Product Inc.), Zoladex™ (Zeneca), Decapeptyl™ (Ipsen Biotech), and Prostap SR™ (Lederle), all of which are licensed for intramuscular use in humans in both Europe and the U.S.A.

Functionalized magnetic nanospheres and microspheres (MMS) are usually formulated by encapsulation of magnetic nanoparticles (e.g., magnetite) into the biodegradable polymeric matrix and are being used evermore for biomedical applications including drug delivery, diagnostic magnetic resonance imaging (MRI), magnetic cell separation, tissue repair, hyperthermia and magnetofection [4-8]. Superparamagnetic iron oxide contrast agents have been approved by FDA for MRI. Commercial iron oxide nanoparticles of maghemite (Endorem^® ^and Resovist^®^) are used as contrast agents in MRI for the diagnosis and exact location determination of brain [9,10] and cardiac infarcts [11], and for the detection of liver lesions and tumors [12], where the magnetic nanoparticles tend to accumulate at higher levels due to the difference in tissue composition and/or endocytotic uptake processes. In a clinical study, it was concluded that intracranial thermotherapy using magnetic nanoparticles can be safely applied on glioblastoma multiforme patients [13]. The toxicity of magnetite encapsulated in PLGA has not been tested, although PLA microspheres containing 10% magnetite were tested in a rat model for treatment of intraspinal glioblastoma by delivering local radiation. After injection of 0.5 mg of microspheres through an intrathecal catheter, all 12 rats survived for 15 months with no apparent side effects or weight loss [14]. Due to their magnetic component, MMS can be locally targeted using externally applied magnetic fields. A promising approach, for example, is the intravascular injection of magnetic particles (ferrofluids) bound to anticancer agents that are then concentrated in the desired area (e.g., a tumor) by an external magnetic field [15]. It might thus be possible to overcome the systemic side effects of many chemotherapeutic agents.

We sought to develop biodegradable sustained release PLGA MMS which could be administered by intravascular injection and which are attracted by external magnetic fields to a specific site [6]. For the desired mode of administration, the size of the MMS must be optimized. The MMS have to be smaller than red blood cells and must be delivered through blood vessels at concentrations which do not embolize (clog) the capillaries. Since the diameter of the smallest blood vessels, the capillaries, is typically 7–8 μm [16], a particle size under 2 μm enables intravascular injection and is also desirable for intramuscular and subcutaneous administration, minimizing possible irritant reactions [17]. On the non-physiological side, particle size also influences both the loading efficiency of the magnetic materials that turn microspheres into MMS and of the therapeutic agents. This is especially important if the loading efficiencies have to be maximized. In general, the larger the particle size, the higher the encapsulation efficiency for drugs and magnetic nanoparticles, as can be explained by the fact that the size increase is related to a relative decrease of the surface area, thus reducing the possibility of drug loss by diffusion during the fabrication procedure [17]. In addition, the magnetic forces required to direct and localize the MMS will increase substantially with decreasing particle size, requiring that the microspheres be large enough to allow the use of reasonably sized magnets [18].

Our work focuses on the effect of process and formulation variables in the preparation of magnetic PLGA microspheres in the specific size range of 1 to 2 μm. A couple of papers describe the use of large PLGA MMS of 20–50 μm [19,20], while Lee et al. prepared magnetic PLGA nanoparticles (90–180 nm) by an emulsification-diffusion method after stabilizing iron oxide nanoparticles by sodium oleate and sodium dodecylbenzenesulfonate [21]. Most recently, Liu et al. have successfully fabricated microspheres containing 45 weight% of oleic acid-coated magnetite using a polymer mixture of PLGA and a diblock copolymer of poly(lactic acid) and polyethylene glycol (PLA-PEG). They used a single emulsion method and loaded the magnetite into the oil phase [22]. To our knowledge, there are no other studies looking at small PLGA MMS of 1–2 μm. In the field of non-magnetic microspheres, however, Muramatsu et al. have prepared uniform PLGA microspheres by forcing the polymer dissolved in methylene chloride through a glass membrane of homogeneous pore size of about 1120 nm [23]. Two other studies examined the preparation conditions of PLA, PLGA and PLA-PEG-PLA particles incorporating DNA, in the size range of 200–700 nm [24] and 1–2 μm [25]. Still another method of preparing PLGA microspheres is acoustic excitation, resulting in over 95% of the spheres having a diameter of 1.0–1.5 μm [26]. But none of these preparations involved the incorporation of magnetic materials.

In the present investigation, both non-magnetic and magnetic microspheres were prepared by a W/O/W double emulsion and solvent evaporation method. Effects of homogenization conditions (speed and time), inner water phase (volume and protein concentration), oil phase (volume, polymer concentration in oil phase and solvent composition), outer water phase (stabilizer concentration) and evaporation speed on particle size were systematically investigated. The size optimized method found during the preparation of non-magnetic microspheres was then applied to the making of MMS by loading water dispersible magnetite coated by polyethylene glycole/polyacrylic acid (PEG/PAA) into the inner water phase.

## Materials and methods

### Materials

PLGA (Lactel^® ^biodegradable polymers) having a lactide/glycolide molar ratio of 85:15 with an intrinsic viscosity of 0.61 dl/g and an average MW of 23,878 (measured by size exclusion chromatography using polystyrene standards) was purchased from Durect Co., U.S.A. Polyvinyl alcohol (PVA, 87~89% hydrolyzed, MW 13,000~23,000) and bovine serum albumin (BSA) were bought from Sigma. Magnetite coated by PEG/PAA (about 8 nm diameter, as can be seen in the sixth figure of results and discussion part) was kindly donated by Materials Modification Inc., U.S.A. Dichloromethane (DCM) and ethyl acetate (EA) were of analytical grade and were purchased from Fisher.

### Preparation of Microspheres

Microspheres were fabricated using a water-in-oil-in-water (w/o/w) double emulsion solvent evaporation method. The optimized method consisted of adding 0.2 ml of a 1 mg/ml BSA solution to a 4 ml mixture of DCM and EA at a ratio of 3 to 1 containing 200 mg of PLGA. A first w/o emulsion was prepared using a homogenizer (Polytron PT10-35; Kinematica, Luzern, Switzerland) in an ice bath at 26,000 rpm for 2.5 min. Fifteen ml of a 1% PVA solution was then poured directly into the primary emulsion and re-emulsified using the same homogenizer under the same conditions for another 2.5 min. This w/o/w emulsion was immediately poured into a beaker containing 85 ml of 1% PVA solution and was stirred in a hood under an overhead propeller for 2 h, allowing the solvent to evaporate. The solidified microspheres were harvested by centrifugation at 2500 rpm for 10 min and washed with distilled water three times.

The processing parameters of homogenization (speed and time), inner water phase (volume and BSA concentration), oil phase (polymer concentration, volume, solvent composition), PVA concentration in outer water phase, and evaporation speed were varied. All trials were run in triplicate.

To prepare the MMS, water dispersible magnetite with a PEG/PAA coating was added to the BSA-containing inner water phase. The same protocol used for the non-magnetic case was then followed.

### Microsphere and Magnetite Nanoparticle Analysis

After gold-palladium coating, the shape and surface morphology of microspheres and MMS were examined using scanning electron microscopy (SEM) (Hitachi S-4500, Tokyo, Japan). To determine the size and morphology of the magnetic nanoparticles, a drop of an aqueous dispersion of magnetite nanoparticles was placed on a formvar-coated copper transmission electron microscopy (TEM) grid (150 mesh, Ted Pella Inc. Redding, CA) and allowed to air-dry.

The zeta potential of the magnetite nanoparticles was determined in a Zetasizer (Malvern Instruments, Malvern, UK) and their magnetic properties on a vibrating sample magnetometer (VSM) (Model 155, Princeton Applied Research).

The microspheres were sized by laser diffraction using a Mastersizer 2000 and the dispersing unit Hydro 2000 MU (Malvern Instruments, Malvern, UK). The average particle size and standard deviation was expressed as the volume mean diameter from 3 different microsphere batches each.

### Determination of Kinematic Viscosity

The kinematic viscosity of PLGA in the DCM/EA (3/1) solvent system and PVA in water of different concentrations was determined using a capillary tube viscometer (Ubbelohde type, Fisher). This method is based on measuring the efflux time of a fixed volume of liquid samples through a capillary tube and then multiplying this time by the viscometer constant. Three determinations per sample were carried out at room temperature and mean value and standard deviation was calculated.

## Results and Discussion

Several microencapsulation methods, such as phase separation spray-drying and emulsion solvent evaporation methods (o/w [21], w/o/w [25], w/o/o [27]), have been developed for the microencapsulation of a wide variety of drugs [28]. Among them, the water-in-oil-in-water (w/o/w) double emulsion solvent evaporation method is probably the most used and has been proven to be advantageous over the single emulsion solvent evaporation method for the encapsulation of highly water soluble compounds [29,30]. In the w/o/w process, the water dissolvable or water dispersible components such as proteins and peptides, and other additives are dissolved or dispersed in an aqueous solution, which is then emulsified in an organic solvent containing the dissolved PLGA. This primary emulsion (w/o) is then dispersed in a second aqueous phase containing a suitable emulsifier and forms a double emulsion (w/o/w). Solid microspheres are collected following the complete removal of the volatile organic phase. All the processing and formulation factors must be controlled since they influence the stability of the emulsion and eventually the particle size, surface morphology, loading efficiency and release pattern of the microspheres [31,32]. In the following text, the influence of these parameters on making small PLGA microspheres will be discussed in detail.

### Influence of homogenization speed on microsphere size

**Figure 1:**
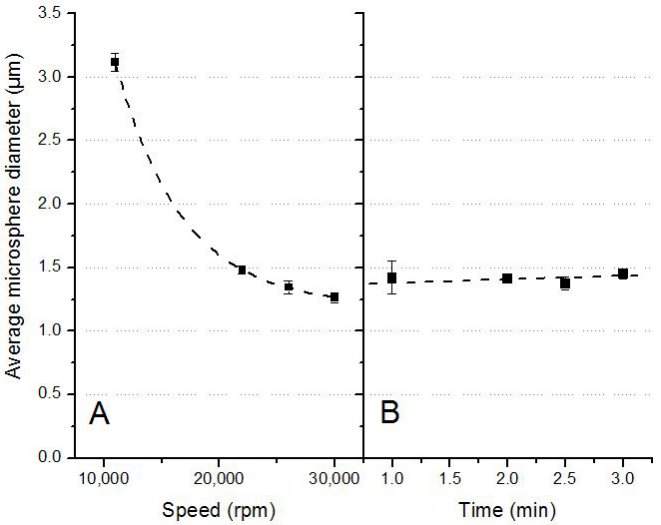
Effect of (A) homogenization speed and (B) time on average microsphere diameter.

According to the literature, the effect of homogenization speed on the size and shape of microspheres is not consistent [21,27,33,34]. In most cases, however, particle size decreases with increasing homogenization speed. In the present study, a dramatic decrease of microsphere size from 3.11 to 1.26 μm occurred when the homogenization speed in both the primary w/o and final w/o/w emulsion increased from 11,000 to 30,000 rpm (Figure 1A).

As the homogenization speed increases, the shear stress increases and the established balance between tangential stress at the droplet interface impacted by the homogenizer and interfacial tension is going to be altered. The larger tangential stress leads to a reduction in droplet size, while the homogenization speed affects the relative viscosity of the emulsion. Typically, the viscosity reduction at a higher rotational speed is responsible for a decrease in particle size [21]. While homogenization speed was found to be the dominant factor for the sizing of microspheres, homogenization times, at least at high homogenization speeds of 26,000 rpm (Figure 1B) did not significantly change the final microsphere size.

### Influence of inner water phase on microsphere size

**Figure 2:**
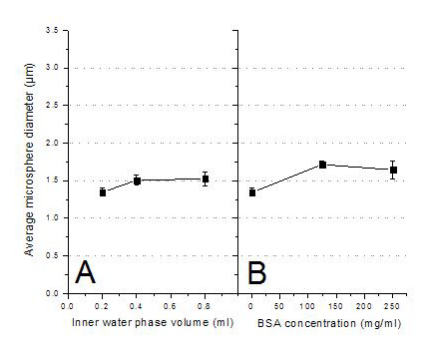
Effect of (A) inner water phase volume and (B) BSA concentration in inner water phase on average microsphere diameter.

An inner water phase volume increase from 0.2 to 0.4 ml resulted in a small microsphere size increase from 1.34 to 1.51 μm (Figure 2A). However, further increasing the inner water phase volume from 0.4 to 0.8 ml showed only negligible size changes. These results are different from those reported by Crotts et al., who showed that the inner water phase volume had a significant effect on final microsphere size [30]. In contrast, their particles were much larger between 20~300 μm. A large inner water volume is especially important when larger amounts of hydrophilic proteins, peptides, or water dispersible components such as the magnetic materials applied in this study need to be encapsulated.

The effect of the protein content, here BSA, in the primary water phase on microsphere size is shown in Figure 2B. The size increased from 1.34 to 1.71 μm with the BSA concentration increase from 1 to 125 mg/ml, but seemed to have reached a maximum at this concentration, as further increases to 250 mg/ml showed no significant changes. Proteins must maintain their intact three-dimensional structure and their chemical integrity during the encapsulation process to allow for delivery of the native protein upon administration. Preparing PLGA microspheres can cause physical and chemical degradation of the to-be-encapsulated protein; therefore the formulation of potent therapeutic proteins into microspheres often requires the use of a carrier protein as a diluent, protective agent and enhancer of encapsulation efficiency. Several stabilization methods have been attempted with variable success. These methods include maximizing the protein concentration in solution, adding a metal (e.g., zinc), adding an additional carrier protein (e.g., albumin), adding a small osmolyte such as trehalose or mannitol, or adding a gelling agent such as gelatin, carboxymethyl cellulose sodium, Arabic gum, or alginate sodium [25,35-37]. In some manufacturing processes, the addition of albumin aided in protecting growth factors from denaturation in organic solvents [38,39]. Our results indicate that BSA has the potential of stabilizing other protein or peptide drugs without significantly changing the size and size distribution of microspheres.

### Influence of oil phase on microsphere size

**Figure 3:**
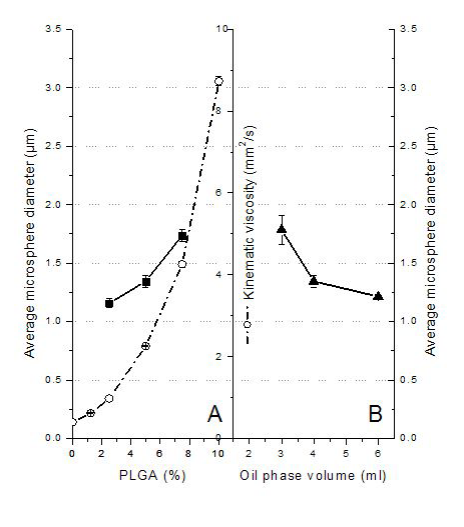
Effect of formulation variables in oil phase. (A) Effect of PLGA concentration in oil phase on average microsphere diameter (▪, y-axis is to the left) and the relationship between PLGA concentration and kinematic viscosity (○, y-axis is to the right). (B) Effect of oil phase volume on average microsphere diameter (▲, y-axis is to the right).

The polymer concentration in the oil phase affects the microsphere size and loading efficiency as well as the release profiles of pharmacological active agents. We found that the size of microspheres increased from 1.16 to 1.74 μm with an increase in PLGA concentration from 2.5 to 7.5% (w/v) in the oil phase (Figure 3A). These results agree with earlier findings using a similar method for the preparation of nanospheres sized 120–180 nm [17,21], microspheres sized 1–3 μm [25], microspheres sized 20–50 μm [40], and even larger microspheres sized 30–210 μm [41]. The increase of PLGA concentrations parallels the viscosity increase of the polymer dissolved in the DCM/EA (3/1) mixture (Figure 3A). The higher the viscosity of the oil phase, the higher the forces that need to be overcome to form fine particles. Highly viscous polymer solutions have been reported to produce microspheres with a dense core, which in turn show decreased initial protein burst release [42]. Furthermore, the viscosity of the polymer solution affects other microsphere properties such as drug content, specific surface area, and porosity, due to its effect on the rate of solvent extraction. The viscosity characterization is thus an important part of the microsphere process development [43].

The effect of oil phase volume on microsphere size was studied by keeping the amount of PLGA constant and increasing the volume of the oil phase from 3.0 to 6.0 ml. The size of microspheres dropped from 1.78 to 1.21 μm (Figure 3B). This may be due to the fact that an increase in the volume of the oil phase generates a less viscous oil phase, from which droplets break off more easily into smaller droplets, thus generating smaller microspheres.

**Figure 4:**
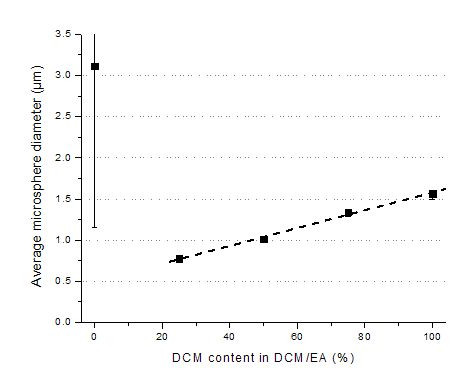
Effect of DCM content in DCM/EA on average microsphere diameter.

The efficiency of encapsulation and the physicochemical properties of microspheres depend to a great extent on the interactions between polymer, drug and solvent. The composition of solvent, including the DCM and EA used here, affect the size, morphology and *in vitro *release profiles due to the speed of polymer precipitation at the polymer/solvent interface [19]. During microsphere preparation, protein molecules are exposed to unfavorable factors. Organic solvents can easily cause aggregation and destabilize the high-grade structure of protein molecules. It is reported that the addition of less toxic EA can result in an increase of glucose oxidase activity in PLA-PEG microspheres, suggesting that EA is a more suitable solvent to preserve the protein activity during microsphere preparation than DCM [37]. For these reasons, we investigated various volume ratios of DCM to EA in the oil phase. The microsphere size increased linearly from 0.78 to 1.56 μm when the percentage of DCM in the mixture increased from 25 to 100% (Figure 4). This may be due to the slower precipitation of the polymer at the particle surface since DCM is less soluble in water and thus diffuses more slowly than EA into the surrounding water phase [44]. However, when pure EA was used as the oil phase a very wide standard deviation was obtained (Figure 4). Presaturating the inner water phase with EA, which was expected to allow for a more defined emulsion preparation, did not improve the size distribution (not shown). Many of the microspheres were distorted and oval in shape. This might be due to a much faster precipitation time of the polymer at the polymer/solvent interface because of EA's lower boiling point and higher water solubility (about 10%). Thus the composition of the solvent plays a key role in the formation of microspheres and affects the solidification time and size of the microspheres [27].

### Influence of PVA concentration and evaporation speed on microsphere size

**Figure 5:**
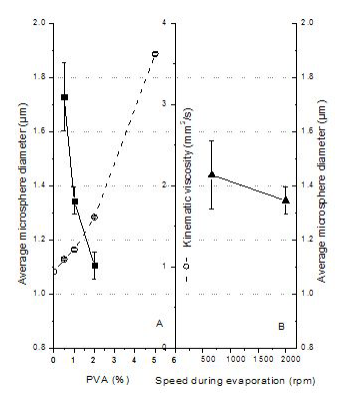
(A) Effect of PVA concentration on average microsphere diameter (▪, y-axis is to the left) and its relationship with the kinematic viscosity (○, y-axis is to the right). (B) Effect of evaporation speed on average microsphere diameter (▲, y-axis is to the right).

A wide range of substances, such as PVA, methyl cellulose, sorbitan monooleate (spans), sodium alginate, gelatin, and sodium dodecyl sulfate, have been used for the stabilization of polymeric microspheres produced by emulsion solvent evaporation techniques [29,45,46]. In this study, PVA was used as the stabilizing and emulsifying agent. When the concentration of PVA was varied from 0.5 and 2% (w/v), the microsphere size decreased (Figure 5A). The effect was probably mainly due to the increasing viscosity of the PVA solution (Figure 5A). During the emulsion formation, the droplets get smaller and smaller under the strong shear stress, while the droplets tend to coalesce again to reduce their surface energy. The presence of surfactant molecules can stabilize the emulsion by forming a protective layer around the droplets thus impeding droplet coalescence and coagulation. Important properties of the emulsifying agent for optimal stabilization of the droplets during microencapsulation are a) a high surface activity (interfacial tension <10 dyn/cm), b) a high viscosity in the external phase, c) an adequate electrical charge, and d) the existence of a film adsorbed on the droplet surface [47]. A good example of choosing these properties correctly is given by Lin et al. who reported that a 1% PVA solution had low interfacial tension towards DCM of ~1.8 dyn/cm and formed a multimolecular film, resulting in better microsphere uniformity [46].

The stabilizer PVA must also be present during the final droplet hardening process, the solvent extraction and evaporation step in order to guarantee a narrow size distribution. The stirring speed at this point, however, does not seem to affect the final microsphere size (Figure 5B).

### Comparison of non-magnetic and magnetic microspheres

**Figure 6:**
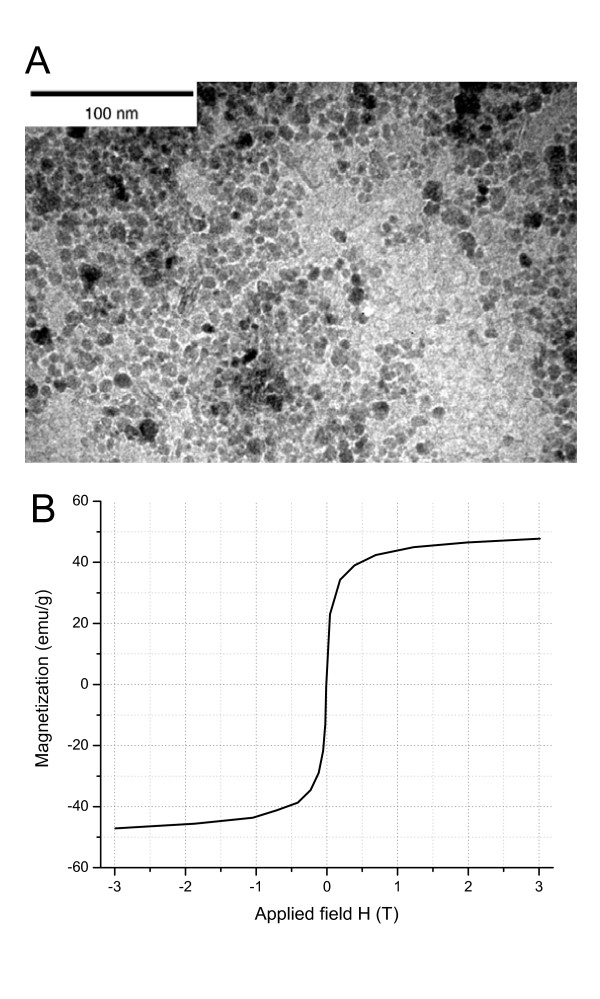
Properties of magnetite nanoparticles with PEG/PAA coating. (A) TEM. (B) Magnetization curve.

Since all optimizations were performed on empty PLGA microspheres, we needed to investigate whether the addition of the magnetic component would alter the size and size distribution of the final MMS. For this purpose we prepared MMS by adding the water dispersible magnetite nanoparticles shown in Figure 6A to the inner water phase. These magnetite (Fe_3_O_4_) particles were of relatively homogeneous size with an average diameter of 8 nm. The magnetization curve in Figure 6B shows that the nanoparticles measured as air-dried powder were superparamagnetic and had a saturation magnetization of 47.8 emu/g. To counteract hydrophobic interactions between particles, including agglomeration, large cluster formation, and strong magnetic dipole-dipole attractions and to stabilize the magnetite nanoparticles, their surface was coated with 6% PEG/PAA. This coating gave the nanoparticles a zeta potential of -34.1 mV and likely helped in stabilization of the suspension and in incorporation of the nanoparticles into the microsphere matrix.

**Figure 7:**
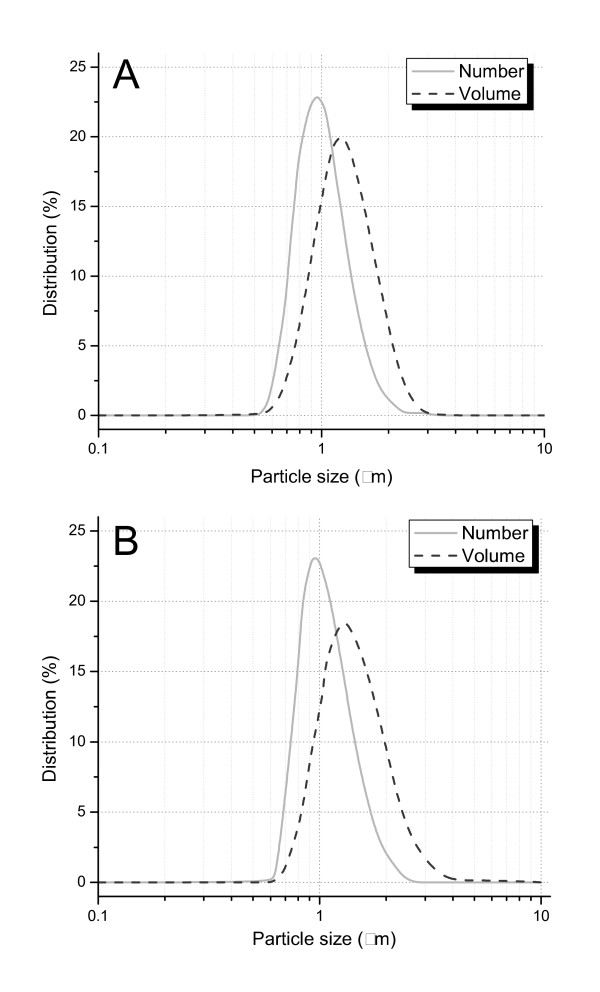
C. Volume and number size distribution of (A) PLGA microspheres and (B) MMS formulated with PEG/PAA coated magnetite.

**Figure 8:**
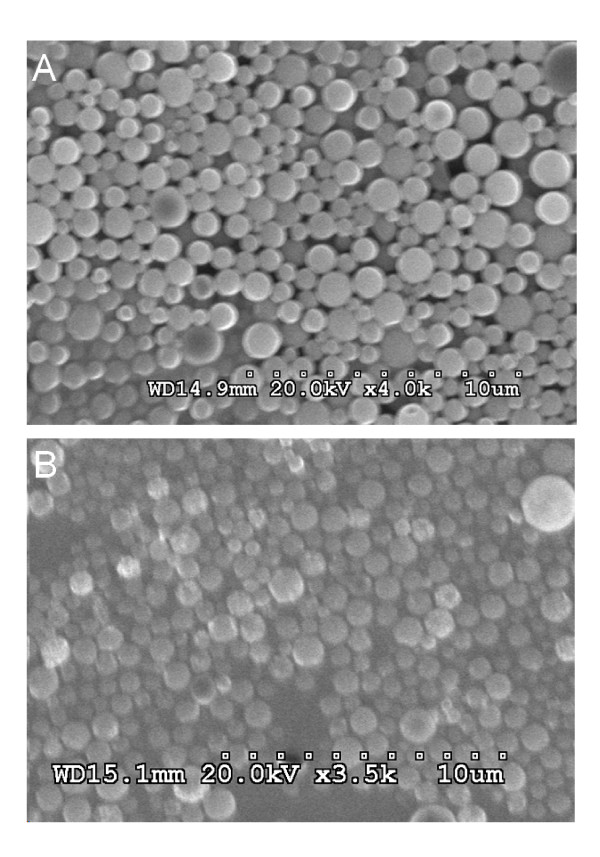
SEM picture of (A) PLGA microspheres and (B) MMS.

**Figure 9:**
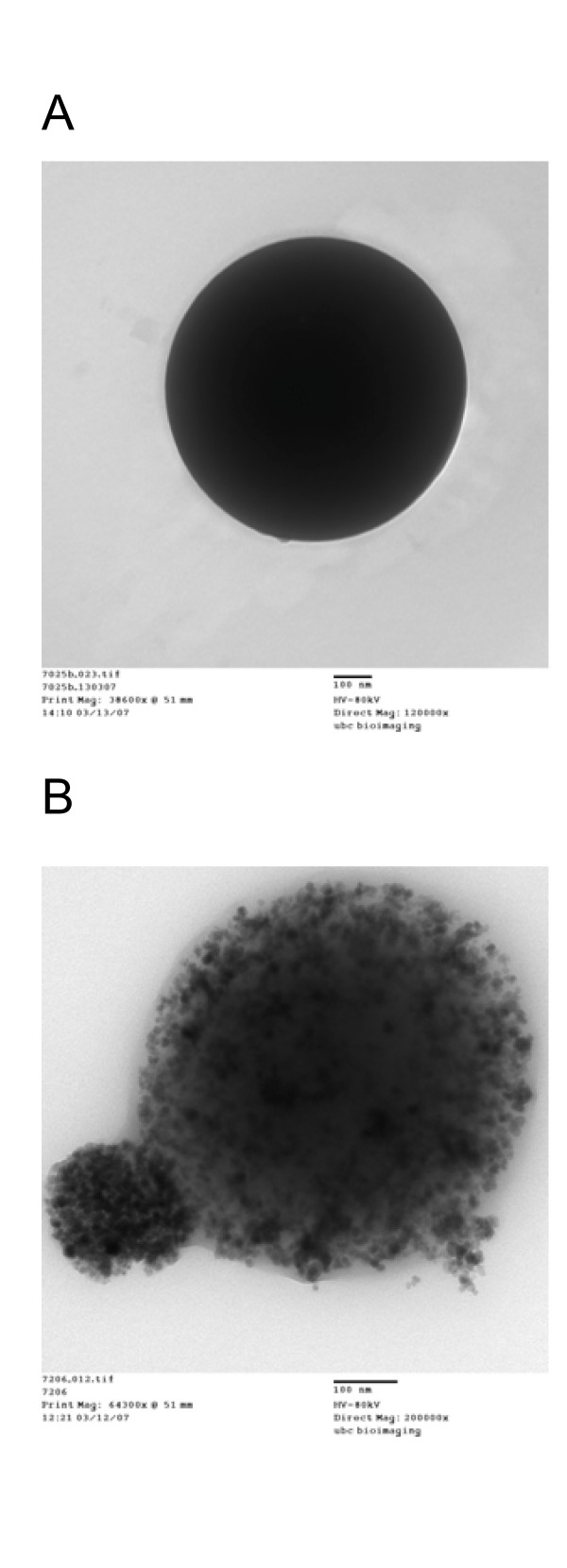
TEM picture of a (A) non-magnetic PLGA microsphere, for comparison purposes, and a (B) MMS to show internal magnetite nanoparticle distribution. The size bar for both pictures is 100 nm long.

Using the optimized conditions found for the non-magnetic microspheres, magnetite microspheres were prepared that contained 13.7 weight% of the magnetite nanoparticles. With a d_50 _size of 1.37 μm, these MMS were statistically indistinguishable from the non-magnetic microspheres with a d_50 _of 1.23 μm. Their size distributions given in Figure 7 were also very close and as narrow as needed for future in vivo applications. SEM pictures (Figure 8) confirm that the size and surface morphology of microspheres were not changed by adding magnetite to the formulation. In combination with information from TEM pictures, it is possible to confirm that the magnetic nanoparticles are dispersed well inside the MMS (Figure 9B). Furthermore, the jagged edges seen by TEM are not seen in the SEM, which means that the magnetic nanoparticles are embedded well by the microspheres' matrix material PLGA.

## Conclusion

Our experiments showed that the size of biodegradable PLGA microspheres can be controlled by modifying the process and formulation variables, in particular the agitation velocity and the parameters that influence the kinematic viscosity of both the oil phase and outer water phase, such as the type and concentration of the oil phase. In addition, the preparation of magnetic PLGA microspheres using the same optimized conditions yielded very similar microspheres in regards of size, size distribution and surface structure. It is important, however, that the magnetic component is stabilized and mixes well with the phase to which it is added. Using nanomagnetite particles coated with PAA/PEG complied with these conditions and did not seem to change the final microsphere properties at all. Such MMS of 1~2 μm have the potential to serve as controlled released drug delivery carriers for intravascular applications, specifically for magnetic targeting of different therapeutic agents with the help of external magnetic fields [6]. Although the encapsulation of pharmacologically active drugs into the PLGA MMS was not investigated in this study, it is expected that highly potent protein or peptide drugs that require dosing in the nano- and microgram amounts will influence size and size distribution of the final microspheres only in a minor way. This is based on the fact that the encapsulation of the protein BSA over a wide concentration range did not alter the size of the final microspheres in this study.

## Declaration of Competing interests

The author(s) declare that they have no competing interests.

## Authors' contributions

All authors contributed equally to the paper.
